# The Role of Mdivi-1 in Reducing Mitochondrial Fission via the NF-κB/JNK/SIRT3 Signaling Pathway in Acute Kidney Injury

**DOI:** 10.33549/physiolres.935445

**Published:** 2025-02-01

**Authors:** Xiao-Yan GOU, Yong LI, Xiao-Ping FAN

**Affiliations:** 1 Department of Radiology, Suining Central Hospital, Suining, Sichuan Province, China

**Keywords:** AKI, Cisplatin, Gentamicin, Iohexol, Mdivi-1

## Abstract

To explore the effects and underlying mechanisms of Mdivi-1 on three common clinical models of acute kidney injury (AKI). Three common AKI cell models were constructed, classified into the control group (human renal tubular epithelial cells [HK-2] cells), the Iohexol group (HK-2 cells treated with Iohexol), the Genta group (HK-2 cells treated with Gentamicin), and the Cis group (HK-2 cells treated with Cisplatin). To explore the optimal protective concentration of Mdivi-1 for each AKI cell model, the experimental design consisted of the following seven groups: the control group (HK-2 cells cultured in medium), three injury groups (HK-2 cells subjected to Iohexol, Gentamicin, or Cisplatin), and the corresponding protection groups (with a certain concentration of Mdivi-1 added to each injury group). Cellular survival and apoptosis, reactive oxygen species (ROS) levels, and the expression of recombinant Sirtuin 3 (SIRT3) in each group were measured. Mitochondrial fission and fusion dynamics in cells were observed under an electron microscope. To explore relevant pathways, the changes in relevant pathway proteins were analyzed through Western blotting. The half maximal inhibitory concentration (IC_50_) values were 150.06 mgI/ml at 6 h in the Iohexol group, 37.88 mg/ml at 24 h in the Gentamicin group, and 13.48 μM at 24 h in the Cisplatin group. Compared with the control group, the three injury groups showed increased cell apoptosis rates and higher expressions of apoptotic proteins in HK-2 cells, with an accompanying decrease in cell migration. After the addition of corresponding concentrations of Mdivi-1, the optimal concentrations were 3 μM in the Iohexo-3 group, 1 μM in the Genta-1 group, and 5 μM in the Cis-5 group, HK-2 cells showed the highest survival rate, reduced apoptosis, decreased mitochondrial ROS and SIRT3 expression, and reduced mitochondrial fission and autophagy when compared with each injury group. Further verification with Western blot analysis after the addition of Mdivi-1 revealed a reduction in the expressions of mitochondrial fission proteins DRP1, Nrf2, SIRT3, Caspase-3, Jun N-terminal Kinase (JNK)/P-JNK, NF-κB, Bcl2, and autophagic protein P62, as well as reduced ROS levels. Mdivi-1 had protective effects on the three common AKI cell models by potentially reducing mitochondrial fission in cells and inhibiting the production of ROS through the mediation of the NF-κB/JNK/SIRT3 signaling pathway, thereby exerting protective effects.

## Introduction

The reported incidence of acute kidney injury (AKI) varies considerably due to differences in observation criteria and the characteristics of study populations. The introduction of the Risk, Injury, Failure, Loss of kidney function and End-stage kidney disease (RIFLE) and the Acute Kidney Injury Network (AKIN) classifications has largely standardized the criteria used in these studies [1,2]. According to the latest international research, the overall incidence of AKI ranges between 2.5 % and 92.2 %, and the mortality rate is 5–80 % [3–5].

AKI refers to a clinical syndrome that occurs due to a rapid decrease in kidney function in a short period of time because of a variety of etiologies. Numerous pharmacological agents are clinically recognized to cause renal damage, and the common drugs include: (1) antibiotics, including Gentamicin from the aminoglycoside class and Ampicillin from the β-lactam class [6]; (2) antitumor drugs such as Cisplatin and Allopurinol [7]; and (3) frequently used contrast agents such as Iohexol and Ioversol [8] When the kidneys are exposed to these drugs, pathological changes in terms of mitochondrial swelling and increased division of proximal renal tubular epithelial cells have been noted. At the molecular level, decreased adenosine triphosphate (ATP) production, increased reactive oxygen species (ROS) and cytochrome C production, disrupted energy metabolism, and cell death have been observed in the kidneys [9–11].

Mitochondria exist in a dynamic equilibrium characterized by constant fission and fusion. Dynamin-related protein 1 (DRP1) is an important regulator of mitochondrial fission, which is mainly recruited to the outer mitochondrial membrane by binding to receptors such as Fis1, mediating the inward constriction of the outer mitochondrial membrane and thereby enabling fission. A large number of current studies have confirmed that DRP1 is mainly involved in inhibiting cell division, migration, and invasion in lung cancer, breast cancer, and neurological diseases [12–15]. Mdivi-1, a novel pharmacological inhibitor of DRP1, has been identified as significant in influencing mitochondrial kinetics, mitophagy, ATP production, immune response, and Ca^2+^ homeostasis [14]. Preliminary studies have demonstrated its promising therapeutic prospects for diseases such as myocardial injury, cerebral ischemia, and neoplasms [16,17].

Mitochondria are highly motile organelles whose functioning is dependent on the equilibrium between fission and fusion. When stimulated by certain pharmacological agents, mitochondria change from elongated filamentous structures to shorter rod-like forms, completing the process of mitochondrial fission. This division results in mitochondria that are smaller in size and more sensitive to membrane depolarization and oxidative stress. Conversely, the fusion of mitochondria allows for enhanced mitochondrial activity, reducing the production of ROS in cells, and promoting sustained energy output.

Recombinant Sirtuin 3 (SIRT3) is localized in the mitochondrial matrix and distributed throughout the organelles. It is involved in several critical functions, such as DNA repair, maintenance of mitochondrial energy homeostasis, deacetylation, and regulation of superoxide release. SIRT3 is highly expressed in the proximal tubule and is a key regulator in the mitochondria of kidney cells [18,19], helping to preserve renal mitochondrial function, encouraging a shift from fission towards fusion in mitochondrial equilibrium, and also regulating microtubule network-dependent trafficking in renal cells.

The specific role of SIRT3 on mitochondria in AKI is not fully understood. SIRT3 has been found to inhibit mitochondrial fission by mediating the Adenosine 5′-monophosphate (AMP)-activated protein kinase (AMPK)-Drp1 pathway [20], further contributing to maintaining mitochondrial homeostasis and cardiomyocyte viability. Another study has highlighted the important role the SIRT3/AMPK pathway plays in the regulation of sepsis-induced AKI through the glycolysis inhibitor 2-deoxy-D-glucose (2-DG) [21].

At present, there are only a few studies that have explored the effects of Mdivi-1 on AKI and its mechanisms. This study aimed at: (1) exploring the effects and mechanism of DRP1 inhibitor Mdivi-1 in three clinical cell models of AKI; and (2) examining the specific role of SIRT3 in the AKI models used in this study.

## Materials and methods

### Main reagents and instruments

The following materials and instruments were used in this study:

human renal tubular epithelial cells (HK-2 cells) (Tongpai Biotechnology Co., Ltd., Shanghai, China);

Culture medium and reagents included 1640 medium, fetal bovine serum (FBS) and 0.25 % Trypsin-EDTA (GIBCO, USA);

Assay kits and compounds included a Cell Counting Kit-8 (CCK8), Cisplatin, Gentamicin, Iohexol (MedChemExpress, USA);

Fixatives and detection kits that were used were glutaraldehyde fixative (for electron microscopy, 2.5%), ANNEXIN V-FITC/PI apoptosis detection kit, ultra-sensitive ECL chemiluminescence reagent A and solution B, TWEEN-20 (Solarbio);

PCR Reagents: SYBR® Premix Ex TaqTM II (Takara); Mdivi-1, DRP1 inhibitor ab144589 (abcam); electron microscopy fixative (Servicebio, Wuhan, China); Total RNA Extraction Kit and Reverse Transcription Kit (Tsingke Biotech Co., Ltd.);

The primer fragments were human SIRT3-F primer fragment GCTCTACACGCAGAACATCG, human SIRT3-R primer fragment AACACAATGTCG-GGCTTCAC, human GAPDH-F primer fragment GGAGTCCACTGGCGTCTTCA, and human GAPDH-R primer fragment GTCATGAGTCCTTCCACGATACC (synthesized by Tsingke Biotech Co., Ltd.);

ROS kit, BCA protein assay kit, SDS-PAGE gel preparation kit (Beyotime, Shanghai, China);

Antibodies included active+pro Caspase-3 Rabbit mAb, ACTB Rabbit mAb (high dilution), HRP Goat Anti-Rabbit IgG (H+L) (Abclonal), DRP1(D8H5) Rabbit mAb #5391, Phospho-SAPK/JNK (Thr183/Tyr185) (81E11), Rabbit mAb #4668, NRF2 (D1Z9C), XP® Rabbit mAb #12721, SIRT3 Ab-AF5135-100 μl (Affinity);

Instruments used were a fluorescence inverted microscope (Carl Zeiss Microscopy GmbH, Axio Scope A1), flow cytometry (BD, January 2007), microplate reader (BioTek, ELX800), PCR gene amplification system (Roche, LightCycler 480), electrophoresis apparatus (PowerPac, DYY-6C), electron microscope sample preparation system (Leica), and a transmission electron microscope (JEOL, JEM-1400PLUS).

### Experimental Method

#### Recovery, passaging, and cryopreservation of human renal tubular epithelial cells (HK-2)

The procedures followed for the recovery of HK-2 cells were as follows: 1) The HK-2 cell cryopreservation solution was removed from the liquid nitrogen tank and thawed in a 37 °C water bath until the ice crystals completely melted. 2) Then, 9 ml of cell culture medium was added, followed by centrifugation at 1000 rpm for 5 min. 3) The supernatant was aspirated and discarded, and 4 ml of medium was added to form a cell suspension. 4) The cell suspension was transferred to a T25 cm^2^ culture flask and placed overnight in a 37 °C incubator with 5 % CO_2_ to allow for cell adherence. 5) The cell culture medium was replaced every other day, and the cells were ready for passaging when approximately 80 % confluency was reached.

Passaging involved the following steps: 1) Cells were passaged when they reached approximately 80 % confluency, typically every two to three days. 2) The waste liquid was aspirated and discarded. The cells were washed with phosphate-buffered saline (PBS). An appropriate amount of 0.25 % trypsin (approximately 0.5–1 ml/T25cm^2^ flask) was added for digestion. When a small quantity of cells began to detach and visible cracks appeared in the adherent cell layer, 2 ml of medium was immediately added to halt the digestion process. 3) The cell suspension was centrifuged at 1000 rpm for 5 min. 4) The supernatant was discarded, and the cell pellet was resuspended with 4 ml of cell culture medium to create a single-cell suspension. 5) The cells were divided into new culture flasks as per a passage ratio of 1:2 to 1:3, and an appropriate amount of medium was added to each flask. The flasks were placed in the CO_2_ incubator for culture.

Cryopreservation was done as per the following procedure: 1) Cells were subjected to digestion following the same protocol as for cell passaging and then centrifuged at 1000 rpm for 5 min. 2) The supernatant was discarded, and pre-chilled cryopreservation solution was added dropwise to create a single-cell suspension. 3) The suspension was aliquoted into cryovials with approximately 1 ml of cell suspension in each vial. 4) The vials were initially placed at −20 °C for 2 h and then transferred to a −80 °C freezer for storage. For extended storage, the vials were moved to liquid nitrogen.

#### Construction of the three AKI cell models

Prior to commencing the cell-level drug screening experiment, the growth curve of HK-2 cells under different seeding densities was measured to determine the optimal number of inoculated cells and culture duration. This was done to ensure a linear correlation between absorbance values and cell numbers.

1. The HK-2 cell growth curve was created as follows: cells were seeded in multiple 96-well plates at densities of 3000, 5000, and 7000 cells/100 μl/well, cultured for 12 h, 24 h, and 48 h, respectively. After the specified incubation periods, the medium was replaced with serum-free 1640 medium, and 10 μl of CCK-8 reagent solution was added to each well. The plates were incubated in the dark for 1.5 h. The absorbance value at 450 nm was measured using a microplate reader. Based on the results, growth curves were plotted, correlating the number of cells with absorbance values (D450).
2. Three groups of AKI models were constructed following these steps: As per the growth curve data, appropriate amounts of cells were seeded into 96-well plates. Once the cells reached the desired confluency, the culture medium in each group was replaced with serum-free medium containing Iohexol, Gentamicin, or Cisplatin at varying concentrations, and the cells were then cultured for 6 h, 12 h, 24 h, and 48 h. The absorbance value at the wavelength of 450 nm was measured with a microplate reader. Cell viability was calculated according to the D450 value, and the curve of cell viability changes caused by different concentrations of drugs was plotted with the concentration of each tested drug as the abscissa and the cell viability as the ordinate. The optimal culture times and the drug concentrations at which the cell survival rate was 50 % were calculated for the three groups of *in vitro* AKI models.

#### Detection of apoptosis

The cell viability of each group was detected using the ANNEXIN V-FITC/PI apoptosis kit as follows: Cells in each group were cultured under their specific conditions. About 1×10^7^ cells were collected by trypsinization, and the harvested cells were suspended with 1× Binding Buffer to achieve a cell density of approximately 1×10^6^/ml. A total of 100 μl cells were obtained and stained with fluorescein isothiocyanate (FITC) and propidium iodide (PI), and analyzed by flow cytometry within one hour.

#### Wound healing assay

The wound healing inserts were placed in a 6-well plate, and cells were inoculated in it at a density of about 3000 cells/50 μl/well. Each group was subjected to its respective conditions. Following treatment, the inserts were removed, and the culture medium was replaced with serum-free medium. The cell growth was observed and photographed under a microscope after culture for 24 h and 48 h.

#### Western blot analysis

For Western blot analysis, cells from the four groups were harvested *via* trypsinization and lysed with radio immunoprecipitation assay (RIPA) lysate containing a 1 % protease inhibitor. The protein-containing supernatant was obtained by centrifugation, and the protein was quantified using a Bicinchoninic Acid (BCA) colorimetric assay. An aliquot of 20 μg of protein was subjected to boiling and denaturation and then used for sodium dodecyl sulfate polyacrylamide gel electrophoresis (SDS-PAGE) gel electrophoresis. The separated proteins were transferred onto a polyvinylidene fluoride (PVDF) membrane.

Non-specific protein binding sites on the membrane were blocked with 5 % non-fat milk powder. The membrane was then incubated with the primary antibody of interest at 4 °C overnight. After this, it was washed three times with Tris buffered saline with Tween-20 (TBST), with each wash lasting five minutes. Subsequently, the corresponding secondary antibody was added, and the membrane was incubated at room temperature for one hour. Luminescence imaging was performed with the ECL Chemiluminescence Development Kit.

#### Screening of Mdivi-1 concentrations

To determine the optimal concentration of Mdivi-1, following the respective treatment for each group in the 96-well plate, Mdivi-1 at concentrations of 0 μM, 2 μM, 4 μM, 6 μM, 8 μM, and 10 μM was added for 6 h, 24 h, and 48 h. After treatment, 10 μl of CCK-8 reagent solution was added to each well, followed by incubation for 1.5 h in the dark to avoid exposure to light. The absorbance at 450 nm was measured using a microplate reader. The optimal protective concentration of Mdivi-1 for each group was determined as per the resulting growth curve.

#### Calculation of ROS levels

Appropriate amounts of cells were plated in a confocal chamber to achieve 80 % confluency. The seven groups of cells were treated according to their respective conditions and washed twice with PBS. Then a solution of 1 ml of 2′,7′-dichlorofluorescin diacetate (DCFH-DA) with a concentration of 10 μM was added and incubated for 20 min in a 37 °C cell culture incubator in the dark. The cells were washed three times with serum-free cell culture medium and photographed under laser confocal microscopy to detect the expression of ROS.

#### Real-time fluorescence quantitative PCR

Total RNA was extracted from the seven groups of cells using an RNA extraction kit. Complementary DNA (cDNA) was synthesized by reverse transcription with the reverse transcription kit, and real-time fluorescence quantitative PCR was performed as per the relevant protocol for the SYBR® Premix Ex TaqTM II reagent (TaKaRa), and the reaction conditions were 95 °C for 5 s followed by annealing at 60 °C for 30 s, repeated for a total of 40 cycles. Expression levels of the genes MMP-13, Col2A1, and GAPDH were calculated using the 2^−ΔΔCt^ method. The primer sequences are shown in Table 1.

#### Electron microscopy photography of cell mitochondria

Following culture under the specified conditions for each group, the cells were harvested and centrifuged to form mung bean-sized pellets, which were fixed in 2 ml of 2.5 % glutaraldehyde for 24 h. The samples were washed three times with 0.1 % phosphate buffer, with each wash lasting 10 min. They were then fixed with 1 % osmium tetroxide for two hours and washed three times with 0.1 % phosphate buffer.

Gradient dehydration was carried out with 50 %, 70 %, 80 %, 90 %, and 100 % ethanol solutions, with each step lasting 15 min. The samples were then subjected to two washes with 100 % acetone for 15 min each time. The dehydration process was followed by embedding in a mixture of acetone and embedding agent in a ratio of 1:1, for one hour, then in a mixture of acetone and embedding agent in a ratio of 1:2 for three hours, and in pure embedding agent overnight.

The samples were then removed, placed into embedding plates, and polymerized at 40 °C and 60 °C for 48 h each. The samples were then cut into thin slices measuring 50–90 nm and stained with uranyl acetate and lead citrate for 15 min. The stained sections were observed and photographed under an electron microscope.

#### Statistical analysis

All experiments were conducted in triplicate, and the results were expressed as the mean ± standard deviation (mean ± SD). Data comparison between two groups was carried out using the independent sample *t*-test, and comparisons among multiple groups were performed using the one-way analysis of variance (ANOVA) or rank sum test based on the homogeneity of variance. ImageJ was used for image analysis and data acquisition, while statistical analyses, including between-group difference analysis and graphical representation were performed with SPSS and GraphPad Prism 8.0 software. Statistical significance was determined with a threshold of P<0.05 (* P<0.05, ** P<0.01, and *** P<0.001).

## Results

### Construction of the AKI model

#### Cell growth curves

**Fig. 1:**
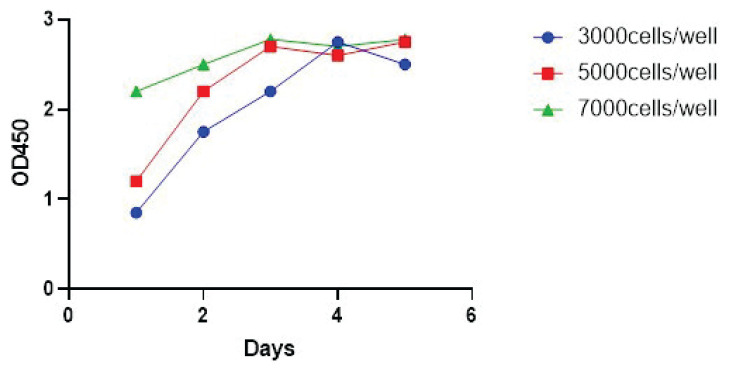
HK-2 cell growth curve. The growth curves for each group of cells were plotted, with the cell growth time as the abscissa and absorbance as the ordinate.

The growth curves for each group of cells were plotted, with the cell growth time as the abscissa and absorbance as the ordinate (Fig. 1). Cells seeded at a density of 7000 cells/well had a slow growth rate or did not show much growth. In contrast, HK-2 cells seeded at a density of 5000 cells/well grew faster than those seeded at 3000 cells/well. The cells entered the logarithmic growth phase by day 2 and reached the plateau phase by days 3 and 4. After this, cell survival decreased due to contact inhibition between cells and insufficient nutrition but recovered to some extent after changing the culture medium and stabilizing around the plateau stage. Based on these findings, an inoculation density of 5000 cells/well and a growth period of 24 h were determined to be optimal for subsequent drug screening experiments.

#### Screening of drug concentrations

**Fig. 2:**
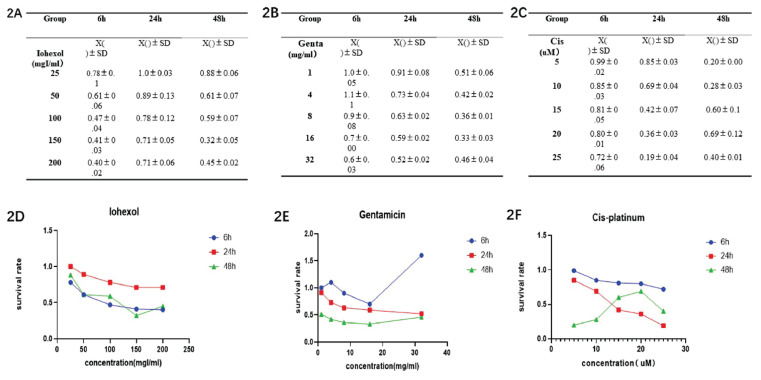
Survival rate of HK-2 cells in each group. (**A, B, C**) the value of cell viability rate at different concentrations in Iohexol, Gentamicin, or Cisplatin group. (**D, E, F**) cell proliferation-toxicity detection curves in each group with the drug concentration used as the abscissa and the cell viability rate as the ordinate.

The relationship between drug concentration and cell viability was analyzed by plotting the cell proliferation-toxicity detection curves (Fig. 2) with the drug concentration used as the abscissa and the cell viability rate (viability rate = [(As-Ab)/(Ac-Ab)]*100 %) as the ordinate. In the Iohexol group, after 6 h of treatment, a strong linear relationship between cell viability and drug concentration was observed, yielding an IC_50_ value of 100 mgI/ml. In the Gentamicin group, there was a better linear relationship between cell viability and drug concentration at 24 h compared with that at 48 h of treatment, with an IC_50_ value of 16 mg/ml. In the Cisplatin group, the cells displayed lower sensitivity to the drug after 6 h of treatment, and the cells developed drug resistance after 48 h of treatment. Therefore, 24 h was determined as the optimal action time for this group, with an IC_50_ value of 25 μM.

#### Apoptosis rate of HK-2 cells in the three cell models

**Fig. 3:**
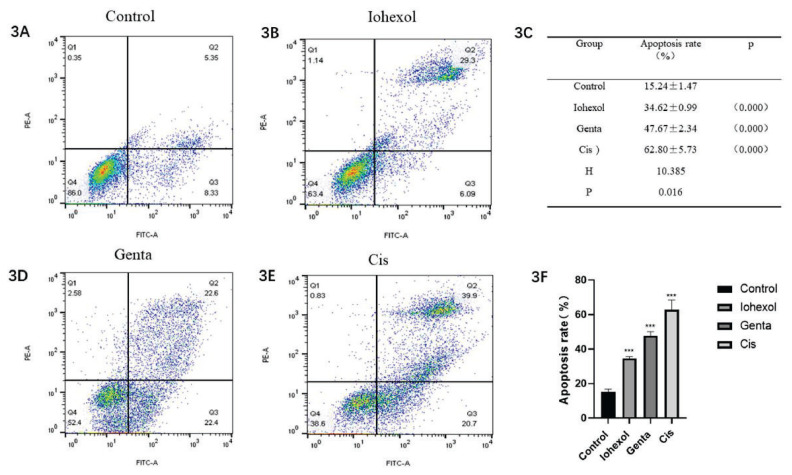
Apoptosis rate of HK-2 cells in each group. (**A, B, D, E**) flow cytometry results in control, Iohexol, Genta, or Cisplatin group. (**C**) value of apoptosis rate. (**F**) bar graph of apoptosis rate.

The following experiments were carried out in each group: control (HK-2+1640 medium); Iohexol group (HK-2 cells + 100 mgI/ml Iohexol); Genta group (HK-2 cells + 16 mg/ml Gentamicin); and Cis group (HK-2 cells + 25 μM Cisplatin). Compared with the control group, which had an apoptosis rate of 15.24±1.47, there was a significant increase in apoptosis across all the other treated groups. Among them, the apoptosis rate was the highest in the Cisplatin group and the lowest in the Iohexol group. There were significant differences in the apoptosis rates of the Iohexol group (34.62±0.99, P=0.000), the Genta group (47.67±2.34, P=0.000), and the Cis group (62.80±5.73, P=0.000) (Fig. 3).

#### Migration rate of HK-2 cells in the three cell models

**Fig. 4:**
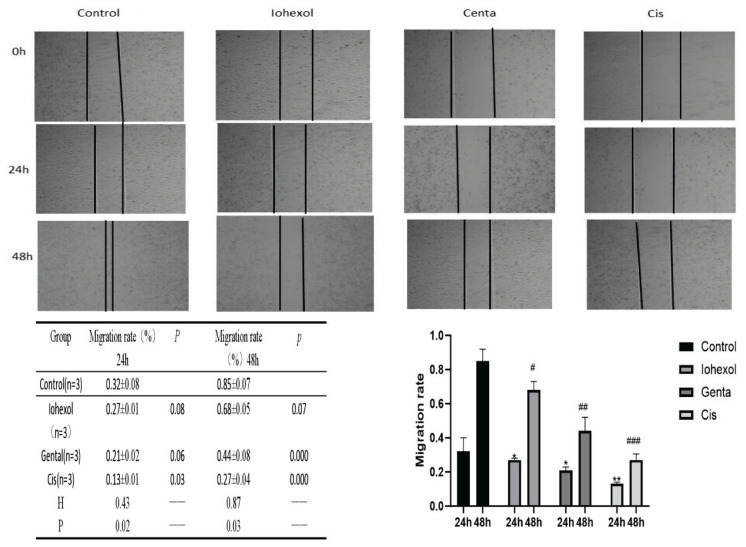
Migration ability of HK-2 cells in each group. Cell growth photographed under a microscope after culture for 24 h and 48 h in control, Iohexol, Genta, or Cisplatin group; value of migration rate; bar graph of migration rate.

The migration rates of HK-2 cells in the three injury groups were significantly lower than those in the control group at both 24 h and 48 h (P<0.5). (At 24 h, Iohexol group vs. control group P=0.34; Genta group vs. control group P=0.27; and Cis group vs. control group P=0.009). At 48 h, compared with the control group, the migration rates of HK-2 cells in the Iohexol, Genta, and Cis groups decreased with statistical significance (Iohexol group vs. control group P=0.29, Genta group vs. control group P=017, Cis group vs. control group P=0.000) (Fig. 4).

#### Screening for the optimal concentration of Mdivi-1

Mdivi-1 had a protective effect across all groups after 6 h of treatment, as indicated by the upward trend in survival rates across all groups. Compared with the Iohexol group, the survival rate of HK-2 cells in the Iohexo-3 group (administered 3 μM of Mdivi-1) was the highest, with a statistically significant difference (P=0.001). Compared with the Genta group, the Genta-1 group (treated with 1 μM of Mdivi-1) had the highest survival rate of HK-2 cells, and the difference was statistically significant (P=0.000). Compared with the Cis injury group, the survival rate of HK-2 cells in the Cis-5 group (treated with 5 μM of Mdivi-1) was the highest, with a significant difference (P=0.000).

For subsequent experiments, the groups were organized as follows: Control group; three injury groups, namely, Iohexol group, Genta group, and Cis group; and three protection groups, namely, Iohexol-3 group, Genta-1 group, and Cis-5 group. The specific treatments for each group were as follows: Control group (treated with normal medium); Iohexol group (treated with 150.06 mgI/ml of Iohexol for 6 h); Genta group (treated with 37.88 mg/m of Gentamicin for 24 h); Cis group (treated with 13.48 μM of Cisplatin for 24 h); Iohexol-3 group (treated with 150.06 mgI/ml of Iohexol for 6 h + 3 μM of Mdivi-1 for 6 h); Genta-1 group (treated with 37.88 mg/m of Gentamicin for 24 h + 1 μM of Mdivi-1 for 6 h); and Cis-5 group (treated with 13.48 μM of Cisplatin for 24 h + 5 μM of Mdivi-1 for 6 h).

#### Real-time fluorescence quantification for detecting the mRNA expression of SIRT3 in HK-2 cells of each group

**Fig. 5:**
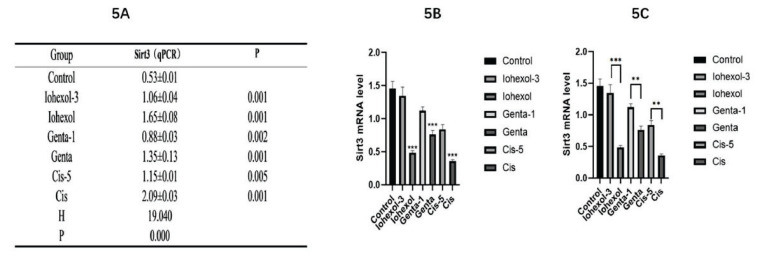
Expression of SIRT3 at the mRNA level in HK-2 cells in each group. (**A**) value of SIRT3 mRNA expression level in control, Iohexol-3 (administered 3 μM of Mdivi-1), Iohexol, Genta-1 (treated with 1 μM of Mdivi-1), Genta, Cisplatin-5 (treated with 5 μM of Mdivi-1), or Cisplatin group. (**B**) bar graph of SIRT3 mRNA expression level in each group. (**C**) comparison of SIRT3 mRNA expression level; * P<0.05, ** P<0.01, *** P<0.001; the injury group was compared with the control group; the Iohexol-3 group was compared with the Iohexol group; the Genta-1 group was compared with the Genta group; and the Cis-5 group was compared with the Cis group; and a rank sum test was performed.

(1) Compared with the control group, the expression of SIRT3 in HK-2 cells of each injury group was significantly decreased (P=0.001). Among the three injury groups, the expression was the lowest in the Cis group and the highest in the Genta group. (2) The expression of SIRT3 in HK-2 cells increased after the addition of an appropriate amount of Mdivi-1 in each injury group. Among them, the expression of SIRT3 was the highest in the Iohexol-3 group and the lowest in the Cis-5 group. Iohexol group vs. Iohexol-3 group (P=0.001); Genta group vs. Genta-1 group (P=0.002); Cis group vs. Cis-5 group (P=0.005), with statistically significant differences (Fig. 5).

#### Determination of ROS levels in HK-2 cells of each group

**Fig. 6:**
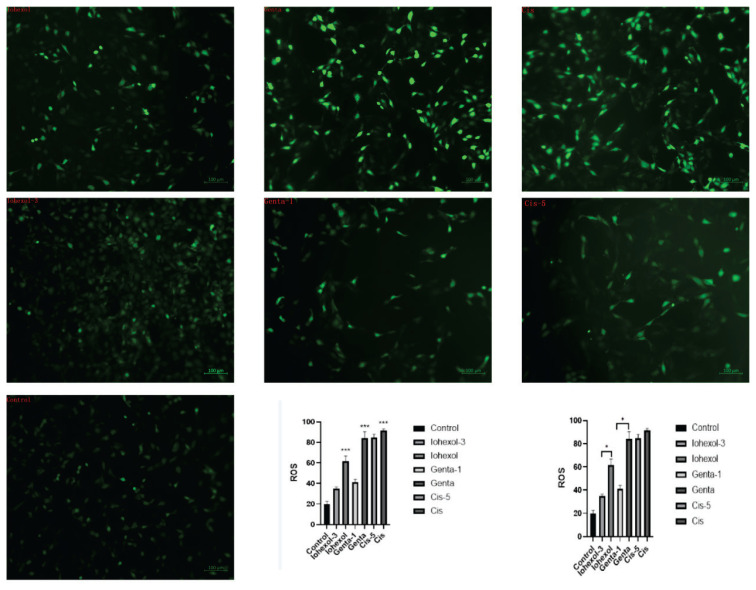
Expression of ROS in HK-2 cells in each group. Laser confocal microscopy photograph in control, Iohexol-3 (administered 3 μM of Mdivi-1), Iohexol, Genta-1 (treated with 1 μM of Mdivi-1), Genta, Cisplatin-5 (treated with 5 μM of Mdivi-1), or Cisplatin group; ROS level in each group; * P<0.05, ** P<0.01, *** P=0.001; the Iohexol, Genta, and Cis groups were compared with the control group; non-parametric rank-sum test was performed. The Iohexol-3 group was compared with the Iohexol group; the Genta-1 group was compared with the Genta group; the Cis-5 group was compared with the Cis group; and a non-parametric rank-sum test was performed.

(1) Compared with the control group (19.90±2.75), ROS production in the mitochondria was significantly increased across all three injury groups (Iohexol: 61.45±5.26, Genta: 84.04±6.38, and Cis groups: 91.61±1.58) (P<0.001). Among them, the Cis group had the highest ROS production, while the Iohexol group had the least. (2) After the addition of an appropriate concentration of Mdivi-1, ROS production was reduced across all injury groups. Among them, there was a significant difference between the Iohexol-3 group (35.02±1.52) and the Iohexol group (P=0.049) and the Genta-1 group (41.29±2.75) and Genta group (P=0.046), whereas no significant difference was observed between the Cis-5 group (85.54±3.49) and the Cis group (Fig. 6).

#### Electron microscopy of mitochondria in HK-2 cells

**Fig. 7:**
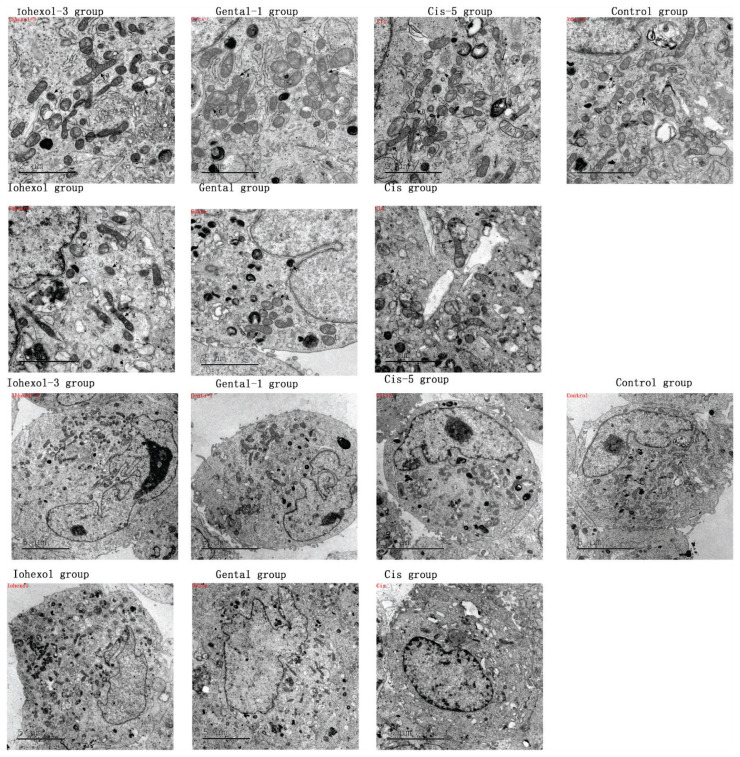
Mitochondrial fission, fusion, and autophagic expression in HK-2 cells in each group. (**A**) Mitochondrial fission in control, Iohexol-3 (administered 3 μM of Mdivi-1), Iohexol, Genta-1 (treated with 1 μM of Mdivi-1), Genta, Cisplatin-5 (treated with 5 μM of Mdivi-1), or Cisplatin group; (**B**) mitochondrial fusion; (**C**) mitophagy.

Following the addition of the three nephrotoxic drugs to HK-2 cells, compared with the normal group (fission: 53 %, fusion: 46.7 %), HK-2 cells in the three groups displayed several notable morphological and functional changes in the mitochondria. There was increased mitochondrial fission, decreased fusion, and increased autophagy. Additionally, some mitochondria appeared swollen, with a loss of cristae and indistinct boundaries: Iohexol group (fission: 62 %, fusion: 37.5 %); Gental group (fission: 67 %, fusion: 32 %); Cis group (fission: 72 %, fusion: 36.5 %). The addition of an appropriate concentration of Mdivi-1 reduced these adverse effects to a certain extent Iohexol-3 group (fission: 57 %, fusion: 42.6 %); Genta-1 group (fission: 60 %, fusion: 39.8 %); Cis-5 group (fission: 68 %, fusion 31 %). (Fig. 7, A: mitochondrial fission; B: mitochondrial fusion; C: mitophagy).

#### Expression of relevant proteins as per Western blot analysis

Compared with the control group, there was a significant decrease in the expression of SIRT3 protein in HK-2 cells in the Iohexol group, Genta group, and Cis group, whereas the expressions of DRP1, Nrf2, Bcl2, NF-κB protein, apoptotic protein Caspase-3, Jun N-terminal Kinase (JNK)/P-JNK protein ratio, and autophagic protein P62 were increased.

Specific comparisons among them demonstrated significant increases in mitochondrial fission protein Drp1 (Iohexol vs. control, Cis vs. control, P=0.000; Genta vs. control, P=0.005), Nrf2 (Iohexol vs. control, Genta vs. control, Cis vs. control, P=0.000), apoptotic protein Caspase-3 (P=0.000), JNK/P-JNK protein ratio (P=0.000), NF-κB (Iohexol vs. control, P=0.001; Genta vs. control, P=0.003; Cis vs. control, P=0.000), Bcl2 (P=0.000), and autophagic protein P62 (P=0.000), all of which were statistically significant.

**Fig. 8:**
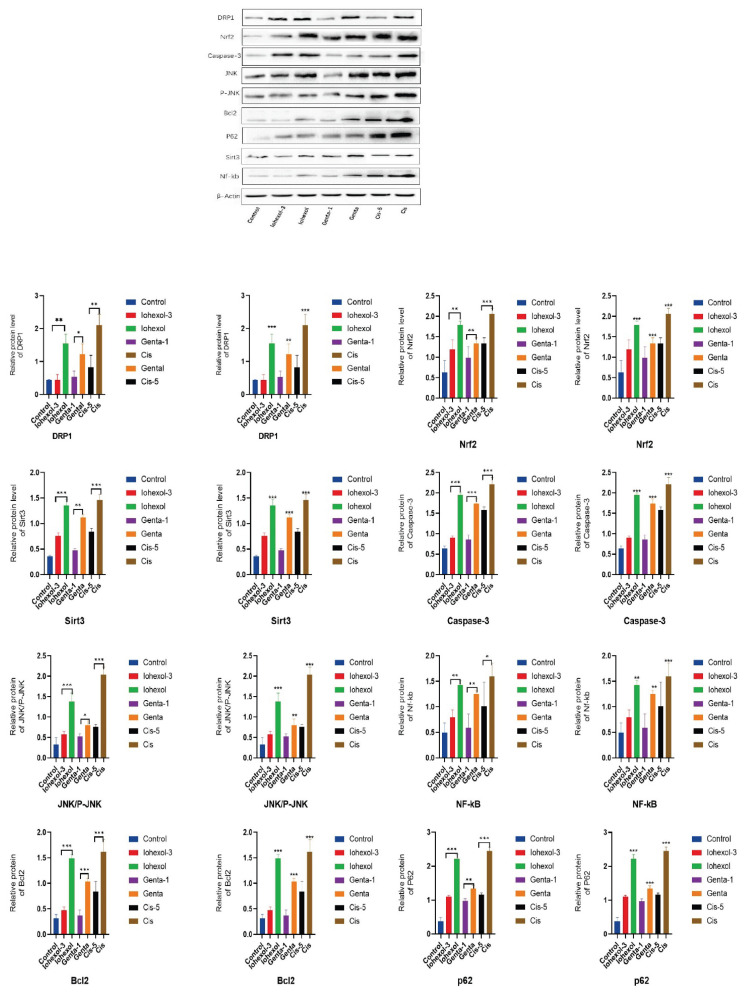
Expression of relevant proteins in each group. Expression of DRP1, Nrf2, SIRT3, Caspase-3, Jun N-terminal Kinase (JNK)/P-JNK, NF-κB, Bcl2, and autophagic protein P62, with β-actin as control in control, Iohexol-3 (administered 3 μM of Mdivi-1), Iohexol, Genta-1 (treated with 1 μM of Mdivi-1), Genta, Cisplatin-5 (treated with 5 μM of Mdivi-1), or Cisplatin group; quantification of expression level in each group.

After treatment with an appropriate concentration of Mdivi-1, the expression of SIRT3 in each protection group increased compared to their respective injury groups, whereas the expression of other relevant proteins decreased. Specifically, there were significant changes in SIRT3 levels (Iohexol-3 vs. Iohexol, P=0.002; Genta-1 vs. Genta, P=0.009; Cis-5 vs. Cis, P=0.000), mitochondrial fission protein DRP1 (Iohexol-3 vs. Iohexol, P=0.007; Genta-1 vs. Genta, P=0.01; Cis-5 vs. Cis, P=0.001), Nrf2 (Iohexol-3 vs. Iohexol, P=0.002; Genta-1 vs. Genta, P=0.01; Cis-5 vs. Cis, P=0.000), apoptotic protein Caspase-3 (P=0.001), JNK/P-JNK protein ratio (Iohexol-3 vs. Iohexol, P=0.007; Genta-1 vs. Genta, P=0.023; Cis-5 vs. Cis, P=0.001), NF-κB (Iohexol-3 vs. Iohexol, P=0.009; Genta-1 vs. Genta, P=0.008; Cis-5 vs. Cis, P=0.016), Bcl2 protein (P=0.001), and autophagic protein P62 (P=0.000), all of which were statistically significant (Table 2, Fig. 8).

## Discussion

In this study, human renal tubular epithelial cells (HK-2 cells) were specifically selected for the investi-gation to minimize the species-specific heterogeneity that can occur when using animal models. Additionally, it has been demonstrated that HK-2 cells can express most of the markers characteristic of human renal tubules [22] thereby providing a closer approximation to primary cultured renal tubular epithelial cells.

Prior to conducting the HK-2 cell experiments, the optimal seeding density and culture duration of HK-2 cells were determined by generating growth curves of HK-2 cells. This approach helped to reduce experimental errors caused by cytotoxicity or suboptimal cell conditions. For the injury model, the concentration corresponding to the half-maximal inhibitory concentration (IC_50_) of each nephrotoxic drug was uniformly selected for the HK-2 cells. The apoptosis rate, migration ability, and expression of the apoptotic protein Caspase-3 in HK-2 cells were assessed to validate whether the AKI model was successfully established.

Our results indicated a significant decrease in the expression of SIRT3 at the mRNA and protein levels upon exposure to the three nephrotoxic drugs. The administration of Mdivi-1 could partially alleviate the decrease in SIRT3. Additionally, Mdivi-1 significantly reduced the mortality rate, mitochondrial fission, ROS production, and the expression of the apoptotic protein Caspase-3 in HK-2 cells across all injury groups.

Electron microscopy further corroborated these findings, revealing that, following the addition of Mdivi-1, mitochondrial fission was reduced and the autophagy of mitochondria in each group was alleviated to a certain extent. These results are also consistent with the expression levels of the autophagic protein P62 observed in the Western blot experiments.

Moreover, alterations in the relevant proteins of the NF-κB/JNK/SIRT3 pathway in the three injury groups were also noted. This trend could be reversed with the addition of an appropriate concentration of Mdivi-1. Consequently, it can be inferred that Mdivi-1 exerted a protective effect against the three common AKIs examined in this study, likely by modulating the NF-κB/JNK/SIRT3 signaling pathway to reduce mitochondrial fission in HK-2 cells.

There are certain limitations to this study: (1) Measurement of other mitochondria-related parameters was not done. A more comprehensive evaluation of overall mitochondrial changes can be achieved by measuring the membrane potential and ATP production in each group of HK-2 cells across different experimental groups. (2) This experiment constitutes only a preliminary investigation at the cellular level, which offers a relatively simplistic perspective. Further validation with animal models is required to corroborate these initial findings. (3) The evaluation of the different impacts of the three nephrotoxic drugs on HK-2 cells was limited to a brief exploration of the changes in three aspects, namely, cell apoptosis, migration ability, and mitochondria. A more detailed exploration of the specific pathways and mechanisms involved would provide a deeper understanding of the effects of the drugs.

## Figures and Tables

**Table 1:** Gene primer sequences.

Target gene	Primer sequence
*SIRT3*	F: GCTCTACACGCAGAACATCG
	R: AACACAATGTCGGGCTTCAC
*GAPDH*	F: GGAGTCCACTGGCGTCTTCA
	R: GTCATGAGTCCTTCCACGATACC

**Table 2:** Determination of relevant proteins in HK-2 cells in each group (mean ± SD).

*Group (n=3)*	Drp1	Nrf2	SIRT3	Caspase-3
*Control*	0.44±0.008	0.63±0.29	0.36±0.02	0.64±0.06
*Iohexol-3*	0.44±0.17 (0.007)	1.19±0.24 (0.002)	0.76±0.06 (0.000)	0.91±0.04 (0.000)
*Iohexol*	1.55±0.28 (0.000)	1.79±0.10 (0.000)	1.35±0.13 (0.000)	1.95±0.05 (0.000)
*Genta-1*	0.53±0.18 (0.01)	0.99±0.27 (0.001)	0.48±0.03 (0.000)	0.86±0.11 (0.000)
*Genta*	1.22±0.31 (0.005)	1.34±0.14 (0.000)	1.12±0.06 (0.000)	1.74±0.12 (0.000)
*Cis-5*	0.82±0.37 (0.001)	1.34±0.14 (0.000)	0.84±0.07 (0.000)	1.58±0.08 (0.000)
*Cis*	2.10±0.33 (0.000)	2.07±0.13 (0.000)	1.46±0.11 (0.000)	2.22±0.16 (0.000)
*F*	12.648	19.818	84.294	115.764
*P*	0.000	0.000	0.000	0.000
*Group (n=3)*	**JNK/P-JNK**	**NF-KB**	**Bcl2**	**P62**
*Control*	0.33±0.17	0.50±0.19	0.32±0.07	0.377±0.11
*Iohexol-3*	0.57±0.08 (0.000)	0.79±0.15 (0.009)	0.48±0.06 (0.000)	1.10±0.04 (0.000)
*Iohexol*	1.38±0.21 (0.000)	1.43±0.09 (0.001)	1.49±0.08 (0.000)	2.22±0.12 (0.000)
*Genta-1*	0.52±0.07 (0.023)	0.59±0.27 (0.008)	0.37±0.11 (0.000)	0.97±0.07 (0.000)
*Genta*	0.81±0.14 (0.001)	1.25±0.18 (0.003)	1.04±0.06 (0.000)	1.33±0.09 (0.000)
*Cis-5*	0.76±0.06 (0.000)	1.01±0.48 (0.016)	0.84±0.20 (0.000)	1.16±0.05 (0.000)
*Cis*	2.04±0.19 (0.001)	1.60±0.26 (0.000)	1.62±0.24 (0.000)	2.45±0.11 (0.0000
*F*	53.548	7.887	44.799	197.863
*P*	0.000	0.001	0.000	0.000

## Abbreviations

β-actin: Beta-actin
CCK8: Cell Counting Kit-8
DRP1: dynamin-related protein 1
FBS: Fetal bovine serum
HK-2: human renal tubular ePithelial
JNK: Jun N-terminal Kinase
PMSF: Phenyl methane sulfonyl fluoride
RT-PCR: Reverse transcription polymerase chain reaction
SIRT3: Recombinant Sirtuin 3
TEMED: Tetramethyl ethylene diamine.
